# Transferrin: Endocytosis and Cell Signaling in Parasitic Protozoa

**DOI:** 10.1155/2015/641392

**Published:** 2015-05-18

**Authors:** Magda Reyes-López, Carolina Piña-Vázquez, Jesús Serrano-Luna

**Affiliations:** Centro de Investigación y Estudios Avanzados del IPN, Apartado Postal 14-740, 07000 México, DF, Mexico

## Abstract

Iron is the fourth most abundant element on Earth and the most abundant metal in the human body. This element is crucial for life because almost all organisms need iron for several biological activities. This is the case with pathogenic organisms, which are at the vanguard in the battle with the human host for iron. The latest regulates Fe concentration through several iron-containing proteins, such as transferrin. The transferrin receptor transports iron to each cell that needs it and maintains it away from pathogens. Parasites have developed several strategies to obtain iron as the expression of specific transferrin receptors localized on plasma membrane, internalized through endocytosis. Signal transduction pathways related to the activation of the receptor have functional importance in proliferation. The study of transferrin receptors and other proteins with action in the signaling networks is important because these proteins could be used as therapeutic targets due to their specificity or to differences with the human counterpart. In this work, we describe proteins that participate in signal transduction processes, especially those that involve transferrin endocytosis, and we compare these processes with those found in* T. brucei*, *T. cruzi*, *Leishmania* spp., and* E. histolytica* parasites.

## 1. Iron

Iron (Fe) is a cofactor of a variety of proteins with important functions for almost all living organisms, prokaryotes, and eukaryotes. Fe is important for several biological processes, such as breathing, oxygen transport, the tricarboxylic acid cycle, gene regulation, and DNA synthesis [1]; however, this element presents high toxicity potential for biological macromolecules [2–6]. Therefore, maintaining cellular Fe concentration requires precise mechanisms to regulate its uptake and storage.

In a normal diet, Fe absorption is approximately 1.5 mg every day. Fe absorption is accomplished through complex mechanisms that are carried out by enterocytes in the upper part of the gut, the duodenum, and the proximal jejunum. The Fe absorbed can be nonhaem Fe or haem Fe. The Fe absorption mechanism involves several import proteins for the two ionic forms of iron, Fe^2+^ and Fe^3+^. Haem Fe from haemoproteins is an important Fe source in omnivores; this is more easily absorbed than nonhaem Fe from vegetables and grains [3, 6].

In the enterocyte, Fe can have several destinations. The Fe destination depends on the iron pool inside the cell. Therefore, Fe can be exported from cells to the circulatory system or can be accumulated inside the cell. For Fe that is exported to the circulatory system, a protein specific for this purpose, ferroportin1, has been identified. Ferroportin1 is a multipass protein found in the basolateral membrane of enterocytes. Once exported by ferroportin1, Fe must be transformed in a process coupled by reoxidation of Fe^2+^ to Fe^3+^ by ferroxidases, such as ceruloplasmin, and followed by the loading of Fe^3+^ onto transferrin (Tf). These proteins are able to regulate iron efflux and consequently iron absorption, because overexpression of ferroportin1 is induced by cellular Fe and is suppressed by hepcidin, which inhibits Fe efflux through binding to and induction of the degradation of ferroportin1 [3, 6].

Cellular Fe metabolism is regulated by Fe itself. When Fe is at low concentrations, iron regulatory proteins 1 and 2 (IRP1 and IRP2), which are components of posttranscriptional regulation, bind to iron responsive elements (IREs) present in the untranslated regions of the mRNAs encoding TfR1, stabilizing it and increasing the number of receptors in the membrane and Fe levels; when Fe levels are high, ferritin synthesis increases, and the receptor mRNA is destabilized, leading to low Fe entry [7–9].

In the blood, Fe bound to Tf, which is the main protein for transporting Fe in plasma, regulates Fe levels in biological fluids. Although Fe is the most essential nutrient for almost all organisms, it has a poor bioavailability and very low solubility and is not found free in nature; therefore, all organisms have invested significant efforts in obtaining Fe.

## 2. Transferrin

Fe exported to the serum is scavenged by Tf, a glycosylated Fe^3+^-binding protein, which is found in blood plasma, lymph, and other body fluids and has as its primary function the transportation of Fe to all cells. Another function of Tf is to keep free Fe at a very low concentration, approximately 10^−18^ M, avoiding the high potential risk of damage and depriving pathogens of Fe, which they require for growth. Tf has an important impact in the defense against infections [10].

Tf is a single polypeptide of about 80 kDa with two homologous lobes (N- and C-terminal) connected by a short center region. Normally, only 30% of the binding sites of the protein are occupied by ferric Fe (Fe^3+^). Tf binds one Fe^3+^ ion in each of the two lobes; the C-terminal lobe binds Fe more tightly and releases it more slowly. Iron binding requires binding of a carbonate/bicarbonate anion in a synergistic way. A complete series of reviews about Tf have been published recently in a special issue of Biochimica et Biophysica Acta (BBA), general subjects entitled Transferrins:Molecular mechanisms of iron transport and disorders 1820(3), 2012.

Serum Tf is synthesized in the liver, central nervous system, testes, ovaries, spleen, mammary glands, and kidneys. Tf is a very highly conserved protein found from bacteria to mammals, including algae [11–14]. Interestingly, Tf is absent in nematodes [15], and unfortunately there is no evidence of the presence of this protein in parasitic protozoa.

## 3. Transferrin Receptors from Mammalian Cells

Cells take up Fe bound to Tf using Tf receptors (TfR); thus, the biological function of the specific receptors is to bind Tf on the cell surface and ingest it. TfRs are a member of the family of tyrosine kinase-linked receptors that possess an intrinsic tyrosine kinase involved in signaling pathways.

Two TfRs have been described in mammals, TfR1 with high-affinity uptake for holoTf and currently the most studied and TfR2 that binds Tf with a 25-fold lower affinity; both TfRs are homodimeric transmembrane glycoproteins that are specific for Fe-loaded Tf (holoTf) [16]. TfR2 shares 45% amino acid sequence identity with TfR1 and plays a critical role in iron homeostasis, with a minor participation in Fe uptake [17].

At low Tf concentrations of <0.3 *μ*mol/L, TfR1 mediates Tf internalization, but at high Tf concentrations, low affinity uptake of holoTf that is not mediated by TfR1 has been observed. TfR2 has been proposed as a receptor that participates in this low affinity uptake, but this receptor is expressed in only a few organs, and the low affinity uptake is found in more cells. Other proteins are responsible for binding and internalize Tf with low affinity, such as the proteoglycans via fluid phase endocytosis in hepatocytes and glyceraldehyde-3-phosphate dehydrogenase (GAPDH) on the macrophage cell surface [10, 18, 19]. It is important to note that several proteins, especially glycolytic enzymes, have been identified with multifunctional properties in both prokaryotic and eukaryotic cells. One of these new functions is to bind Tf in order to obtain Fe [20, 21].

TfR1 is a homodimer linked by two disulfide bridges with a molecular mass of 190 kDa. The receptor is formed by 3 domains: transmembrane, cytoplasmic, and extracellular, which is the larger one and contains the Tf binding site [9, 10, 22].

Tf internalized by both high and low affinity uptake receptors is transported to early endosomes as described below. Once Tf binds to the extracellular domain of the TfR on the plasma membrane, it changes conformation and dimerizes, and this change allows the activation of kinase activity, and it becomes phosphorylated. The Tf-TfR complex enters the endocytic pathway via endocytosis mediated by clathrin-coated pits. The action of dynamin is crucial for the fission of pits from the plasma membrane and the formation of coated vesicles. The Tf-TfR complex is transported to a unique endosomal compartment where acidification (pH lower than 5.6) leads to the release of ferric iron in 2-3 min [3, 23].

Successively, Tf without Fe (apoTf) bound to the receptor is transported to the plasma membrane via endocytic recycling compartments (ERC). The receptor becomes dephosphorylated, and apoTf is released outside the cell in order to bind new Fe [16, 17, 24]. All proteins involved in signal transduction depend on receptor activation produced by the binding of the ligand [25, 26].

Tf, through the TfR, acts as a growth factor. Therefore, its function is important for the regulation of embryogenesis, cell growth, motility, proliferation, differentiation, glucose metabolism, and apoptosis and is determined by its trafficking through the endosomal pathway [27].

In the same way as in mammalian cells, TfR from parasites increases the uptake of Tf, and in consequence of Fe, through the expression of a specific receptor or binding protein that is associated with the course of the infection. TfRs are very important determinants of virulence in pathogens, and depending from the environment where the infection takes place the receptor is or is not expressed. In this sense, parasites in blood vessels express TfR to bind Tf present in this environment, even more in other environments where the presence of this receptor will not be expected: like inside cells, some parasites express TfR, such as* Leishmania,* and it has been demonstrated that this parasite developed strategies to increase the presence of Tf inside the parasitophorous vacuole where* Leishmania* lives. These elaborated systems of Fe obtained by TfR expression ensure their success as parasites, host colonization, and the establishment of the infection.

## 4. TfR in Protozoan Parasitic Organisms

A successful infection by pathogens relies on the host colonization. Colonization depends on the availability of nutrients and growth factors, such as Fe. The relationship between parasitic organisms and their hosts is especially complex, because the hosts must obtain Fe from the diet and fulfil their own Fe needs and at the same time sequester this nutrient away from invading pathogens. Tf is the iron-containing protein that fulfils this activity, but protozoan parasites do not express Tf or Tf-like proteins that help them to acquire Fe. As these organisms are highly dependent on a plentiful supply of host Fe, they have developed mechanisms to acquire this metal by multiple and divergent pathways or steal it from host Fe deposition sites [1, 28].

These mechanisms include the secretion of specific Tf proteases, the presence of reductases that capture host Fe-containing proteins, or through specific TfR or Tf binding proteins [29]. The presence of TfR to piracy Fe must be effective enough to ensure parasites pathogenic potential and proliferation.

TfRs have been described in* Plasmodium *spp. [30, 31],* Tritrichomonas* [32], trypanosomatids such as* Trypanosoma brucei* [33],* Trypanosoma cruzi *[34] and* Leishmania *spp. [35], and the amoeba* Entamoeba histolytica* [36]. It is remarkable that these parasites express receptors that function similarly and recognize the same carrier proteins as the mammalian cell, even though some of them are structurally different and others utilise a completely different mechanism despite their similar function.

The use of specific receptors to obtain growth factors or nutrients ensures temporal prolongation of signal transduction initiated upon ligand binding at the plasma membrane and continued after internalization. In this review, the TfRs from parasites, mainly those with signal transduction studies, have been included and compared with what is known in mammalian cells.

*Trypanosoma brucei*. African trypanosomiasis is caused by the protozoan* Trypanosoma brucei. *This is a very important disease, because millions of people are at risk of infection and because current chemotherapies are toxic [37, 38].

The cell cycle of this protozoan consists of two general stages, one in humans and the other in the insect vector, the tsetse fly (genus* Glossina*). The initial infection is in the gut of the fly (procyclic stage). The infection travels to the salivary glands, and the parasite differentiates into the epimastigote stage and then to the metacyclic stage. This is the stage in which the parasite is injected into the mammalian host. The parasite lives within the mammalian bloodstream as its slender form, and when it is necessary, the parasite transforms into the stumpy form as a prelude to another insect infection [39]. In the bloodstream stage, this parasite is confronted by severe conditions of Fe scarcity. The sole source of iron provided by the host is available as Tf.

Therefore, bloodstream forms of* T. brucei* express a Tf receptor (*Tb*TfR) that mediates Tf endocytosis at the plasma membrane; this receptor has already been identified and is structurally completely different from the host TfR [33, 35, 40, 41].* Tb*TfR is formed by a complex of the proteins encoded by two expression site-associated genes, ESAG6 and ESAG7. ESAG6 has a glycosylphosphatidylinositol (GPI) anchor that attaches the receptor to the plasma membrane. The binding of Tf requires the association of both ESAG proteins [42, 43]. ESAGs are cotranscribed with the gene encoding the variant surface glycoprotein (VSG) of the surface coat of the parasite. VSGs display the adaptation mechanism of antigenic variation [44]. This process allows the development of sustained infections.

Most trypanosomatid protozoa have a specific structure that allows uptake of nutrients at a specific membrane site, named the flagellar pocket. This is a cell membrane invagination from which the flagellum emerges. In this structure, endocytosis of Tf takes place [45]. The molecular mechanism for Tf internalization is through a dissimilar mechanism to that observed in mammals.

When Tf binds to the* Tb*TfR anchored to membrane by the GPI tail, it is internalized by clathrin-dependent endocytosis. The low pH of the endosome allows the release of Fe from Tf. Tf at this pH has a low affinity for the* Tb*TfR [40] and is released and transported to lysosomes for degradation by the action of the* T. brucei* cysteine-protease rhodesain or cathepsin L activity (*Tb*CATL) and* Tb*CATB (*T. brucei* cathepsin B) [33, 46].* Tb*TfR is recycled to the cell surface to bind new Tf and the Fe associated with it [41, 47]. The main difference with mammalian cells is that the Tf is maintained attached to the receptor and transported to the extracellular medium in order to bind new Fe. However, the degradation of Tf in parasites could be for nutritional purposes.

The TfR is of great importance for parasite adaptability and for the ability to colonize several hosts. Because* Tb*TfR has a low-specificity for Tf, the parasite can use Tf from different sources providing the parasite the opportunity to increase its number of hosts, including humans and cattle [44, 48]. The use of Tf from different sources is important for the diversification of species that can be infected by parasite organisms.

*Trypanosoma cruzi*.* T. cruzi* is an intracellular protozoan, the causal agent of South American trypanosomiasis or Chagas disease, which infects 8 million people in Latin America [49].

Similar to the* T. brucei* parasite,* T. cruzi* infects humans and invertebrates hosts during defined stages of the life cycle. The invertebrate host is the triatomine bug that ingests trypomastigotes present in the bloodstream of an infected mammalian host when it feeds. In the gut of the vector, the parasites transform into epimastigotes and migrate to the posterior gut. Then, they transform into infective trypomastigotes, and the vector inoculates them subcutaneously into the mammalian host with infective feces. Once inside, parasites invade several kinds of cells through a lysosome-mediated mechanism, differentiating into amastigotes that replicate and transform into trypomastigotes causing host cell lysis that releases parasites into the bloodstream; the parasites are then capable of invading other cells or infecting vectors that make a meal of the host [50].

*T. cruzi *amastigotes growing in cell-free medium and epimastigotes require high concentrations of Fe to survive, and curiously in these stages, they are able to obtain Fe from human Tf. Amastigotes present specific TfR in the flagellar pocket [51] that are not present in the trypomastigote form.* Tc*TfR presents structural homology with human TfR, with a 200 kDa molecular mass, and Tf is internalized by receptor-mediated endocytosis. However, in the epimastigote stage of the life cycle, the parasite ingests Tf at the cytostome/cytopharynx through a TfR [52]. This structure is a membrane invagination that is similar to the flagellar pocket, but the cytostome reaches deeply into the cytoplasm in the direction of the nucleus. The Tf receptor-mediated uptake is through small, uncoated vesicles to the reservosomes [52]. The participation of uncoated vesicles suggests that the TfR is not recycled to the membrane [34, 53].

Despite the fact that this parasite has clathrin that could participate in endocytosis [54, 55], morphological studies have demonstrated that Tf internalization is carried out through a clathrin-independent and cholesterol-dependent endocytosis pathway. This pathway was identified by the utilization of specific inhibitors of endocytic pathways. Clathrin-dependent internalization similar to that of* T. brucei* should not be excluded; the cholesterol-dependent pathway could be a secondary endocytic process, because inhibition of this pathway did not reduce cell proliferation [34, 56]. Correct Tf internalization requires the association of the cytostome with the flagellar complex in a way that is not well understood [57]. It would be interesting to know whether this route of Tf entry is constitutive or if it depends on the stage of the life cycle of the parasites, because the trypomastigote is the natural form that would confront human Tf.

*Leishmania spp*. The leishmaniases and fatal visceral leishmaniases are diseases with a large spectrum of clinical symptoms in mammals and are caused by at least 20 pathogenic obligate intracellular species that include* Leishmania major*,* L. infantum*,* L. braziliensis, L. mexicana*,* L. amazoniensis*,* L. tropica*, and* L. donovani*. Approximately 2 million new cases occur every year with an estimated 150 million people infected worldwide [58, 59].

The infection starts with the bite of an infected sand fly (dipteran insects) that inoculates metacyclic promastigotes (infective form) into a mammalian host. After being phagocytosed by macrophages, the parasites are found inside parasitophorous vacuoles (PVs); these acidic structures are similar to phagolysosomes and contain certain lysosomal enzymes. Inside the PVs, promastigotes transform into amastigotes. The parasites replicate and induce cell lysis; released parasites can be phagocytosed by adjacent macrophages or infect the surrounding cells. Sandflies become infected by ingesting infected cells during blood meals; amastigotes transform into promastigotes in the gut and then migrate to the proboscis for a new round of infection [60, 61].

Once the parasites are released and before the promastigotes are phagocytosed, they could be encountering Tf from the bloodstream; thus, the expression of a specific receptor would be useful. The presence of a specific and saturable TfR similar to the mammalian TfR was described in promastigotes. The TfRs of* L. infantum* (*Li*TfR) and* L. major *(*Lm*TfR) were described as an integral membrane monomeric glycoprotein of 70 kDa that is structurally different from the mammalian receptor [35]. In both developmental forms of* L. chagasi*, promastigotes and amastigotes, the binding of Tf is through nonspecific and saturable Tf binding proteins [62]. Unfortunately, the Tf endocytic process used by this parasite has not been described.

*Leishmania* amastigotes are usually the form internalized by the mammalian host cell, but in the case of* L. amazoniensis* promastigotes [60], they can also be internalized and then be able to survive and establish within PVs. Promastigotes and amastigotes inside the PV face conditions that include extremely restricted access to essential Fe, and* Leishmania* parasites have developed several strategies for surviving inside the mammalian host. One strategy consists of fusion of the PV with several individual vacuoles and fusion of the resultant vacuoles with compartments of the endolysosomal system. This was discovered because proteins specific for each of the vacuoles are found associated with PVs [60, 63]. In this form on the tenth day of infection, the mammalian Tf-TfR complex normally found in early and recycled endosomes is associated with the PV [64]; furthermore, Tf was found to be delivered to PV and then endocytosed by intracellular amastigotes, so it could be possible that infection time enhances the endosomal delivery to the PV [63]. The iron obtaining mechanisms could be different depending on the* Leishmania* species, because in* L. mexicana*-infected macrophages Tf was not present in the PV [64]; however, amastigotes survive in this environment suggesting the presence of an alternative iron source.

Leishmanial infection would also affect the TfR recycling regulation on macrophages [64], resulting in a Tf disorder where Tf could reach other late or lysosomal compartments and probably be transported to PVs.

Once the Tf is endocytosed by* Leishmania* intracellular parasites, it is delivered to the cysteine proteinase-rich compartments, where this protein is degraded [63].

Another strategy to obtain iron inside the acidic PV from the Tf-TfR complex could present a similar behaviour as in the endosomes; that is, the Tf loses affinity for Fe and remains attached to the receptor and iron release could be facilitated via Tf degradation by cysteine proteases secreted by living amastigotes or released by the lysis of the dead parasites [63]. Then, this element is transported by means of a parasite-associated or -secreted reductase like the Leishmanial iron transporter 1, LIT1, which plays an important role in Fe acquisition by converting Fe^3+^ into Fe^2+^ for transmembrane transport and allowing Fe to be internalized by the parasite [32, 61, 65]. This iron transporter provides enough Fe for the intracellular growth and virulence of* Leishmania*.

*Entamoeba histolytica*.* E. histolytica* is a parasitic protozoan of humans. It causes amoebiasis, a global disease characterized by dysentery and intestinal ulcer production. Under certain conditions, the parasite is able to invade the liver, lungs, and brain.* E. histolytica* infects 500 million people, causes disease in 50 million people, and causes death in 100,000 people each year [66].

*E. histolytica *has an absolute necessity of Fe. This need in the bowel can be sustained by bacteria or phagocytosed red blood cells or through endocytosis of Fe-containing proteins from the host. During invasive amoebiasis, the Fe source is Tf in the bloodstream and liver; in this organ, the use of ferritin, an Fe-storage protein, would be useful for the parasite.

The amoeba has developed two specific mechanisms for obtaining Fe from Tf [36, 67] to ensure it obtains the Fe needed for colonization and infection. One mechanism is by receptor-independent internalization which is active at high Tf concentrations (micromolar range) [68], and at low Tf concentrations between 1.1 and 5.6 nM, the internalization is through specific* Eh*Tfbps of 70, 96, and 140 kDa molecular mass as previously described [67]. Similar to mammalian cells, Tf is internalized by two mechanisms with differing affinity depending from the Tf concentration. More studies must be performed to determine the relationship between the presence of a specific receptor with low or high binding affinity for Tf and Fe necessity.

The* Eh*Tfbps identified present structural homology with the human TfR, because this is recognized with an anti-human TfR antibody. Similarly,* T. cruzi* Tf receptor is recognized with the same antibody, while* Tb*TfRs are different proteins not recognized by human TfR antibodies.* Eh*Tfbps form a complex with holoTf and are endocytosed with the participation of clathrin [67, 69, 70]. Tf is transported into the endolysosomal system (unpublished results).

The 96 kDa* Eh*Tfbp was identified as enzyme acetaldehyde/alcohol dehydrogenase-2 (*Eh*ADH2) [67]. The other* Eh*Tfbps have not been identified (70 and 140 kDa). This enzyme is essential for the growth and survival of* E. histolytica* and allows the parasite to obtain energy through glucose fermentation and to convert acetyl-CoA into ethanol. This enzyme binds extracellular matrix proteins and is found on the cell surface and in the cytoplasm [71]. This protein may be participating in binding Fe from Tf, because in the absence of Fe (apoTf), it does not bind Tf.

In this parasite, other glycolytic enzymes have been described with several functions, such as enolase, which interacts with the activity of the* Eh*meth enzyme that catalyzes DNA methylation [72].

As previously described, surface-localized GAPDH has a novel function with TfR in human and murine macrophage cell lines [19]. GAPDH is capable of interacting with Fe bound to Tf. This enzyme forms a complex with Tf and is taken to early endosomes. The same enzyme with a similar function was reported in* Staphylococcus aureus* and* Staphylococcus epidermidis*, bacteria capable of removing Fe from Tf via a receptor-mediated process [73]. Interestingly, these enzymes bind proteins from the extracellular matrix, like fibronectin and laminin, in addition to plasminogen, plasmin, lysozyme, myosin, and actin [19, 73, 74]. These proteins have been termed moonlighting or multifunctional proteins [21] due to their ability to have more than one function. Other glycolytic enzymes with multiple functions unrelated to their role in glycolysis are *α*-enolase, lactate dehydrogenase, and hexokinase [20].

The life cycle of protozoan parasites suggests why these organisms require an extensive network of cell surface signaling molecules. For example,* E. histolytica* has to compete with bacteria for Fe, other nutrients, and space in the intestinal microenvironment, and intra- or extracellular trypanosomatids in the mammalian host must obtain Fe and other nutrients that are present in very low concentrations. In addition, these parasites must sense several stressors to regulate the different stages of their life cycle to evade host defenses or control their invasive behaviour. Upon invasion, parasites continue to face a battery of challenges that require the ability to adhere and obtain sufficient nutrients. The survival of these parasites within their host requires a profound ability to sense and respond to environmental challenges, and utilization of an extensive signaling network may therefore be very useful.

## 5. Internalization Pathway and Signal Transduction Pathway

Despite the fact that there is much information about TfR signaling pathways in mammalian cells, very little information is available in protozoan parasites, despite the fact that this pathway regulates proliferation and cell growth. In describing these pathways, the emphasis will be placed on the Tf-TfR complex in mammalian cells and the way in which information travels from the cell surface to the cytosol in comparison with that observed in protozoa.

**Figure 1:**
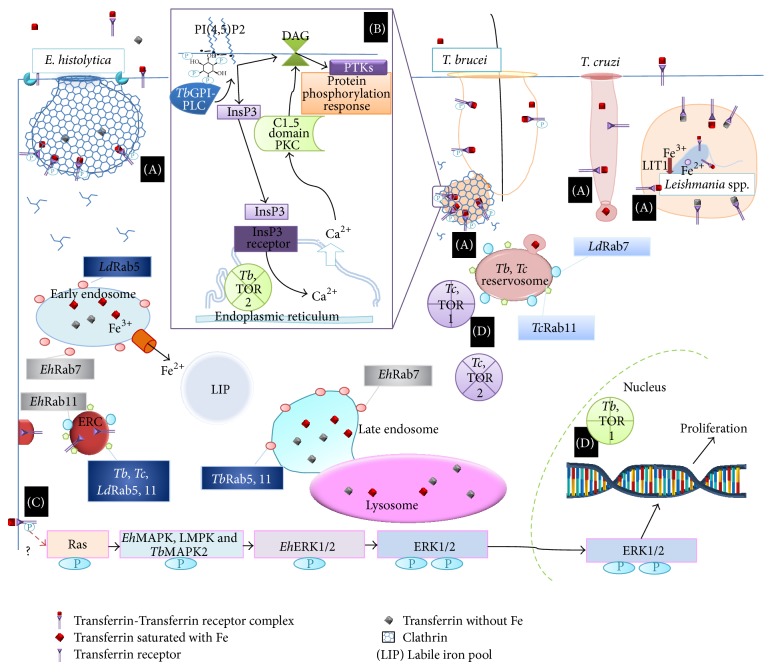
Transferrin endocytosis and signaling pathways in protozoan parasites. (A) Trafficking and insertion of membrane vesicles. The Tf-TfR complex is endocytosed in clathrin coated vesicles in* T. brucei* and* E. histolytica* but in noncoated vesicles in* T. cruzi*. The monomeric G proteins, Rabs, play a role in controlling the trafficking and insertion of new vesicles into endosomes or with endocytic recycling endosomes (ERCs) that recycle the receptor in* T. brucei* and* Leishmania*; in the case of* T. cruzi*, the receptor is not recycled back to the membrane. (B) Inositol-1,4,5-triphosphate and diacylglycerol signaling pathway. In* T. brucei* and* Leishmania*, TfR activation stimulates the formation of InsP3 and DAG through the action of GPI-PLC. Insp3 produces Ca^2+^ release from the endoplasmic reticulum to stimulate cell proliferation. Ca^2+^ in the cytoplasm binds to calmodulin (CaM) and translocates into the nucleus. DAG activates PKC, which then phosphorylates proteins that generate a specific response. (C) MAPK signaling pathway. TfR activated by Tf binding results in phosphorylation of MAPK, which has a central role in cell proliferation, and phosphorylation of the ERK1/2 kinases, which then translocate into the nucleus to activate transcription factors. These types of kinases are described in* E. histolytica*,* T. brucei,* and* Leishmania*. (D) Growth factor signaling pathway through TOR. Active PI3K takes information to TOR complexes that regulate protein synthesis by phosphorylation. TOR kinase functions are well conserved in eukaryotes with some differences in cellular localization in* T. brucei* and* T. cruzi*.

Tf trafficking of information inside the cell and initiation of several signaling pathways are very well defined in mammalian cells: (1) trafficking and insertion of membrane vesicles, (2) inositol-1,4,5-triphosphate and diacylglycerol signaling pathway, (3) MAPK signaling pathway, and (4) growth factors signaling pathway (Figure 1).

### 5.1. Trafficking and Insertion of Membrane Vesicles

The initial signal propagation is in the plasma membrane for endocytosis and then through the endocytic compartments [27, 75–79]. Endocytosis of the Tf-TfR complex is regulated by the concentration of phosphatidylinositol 4,5-bisphosphate (PI4,5-P_2_) in the plasma membrane, which induces the recruitment of clathrin and its adaptor protein AP-2 [80, 81]. The processes of invagination and scission of the clathrin-coated pits are regulated by actin and actin-binding proteins [82–85] that increase the affinity for the dynamin 2 GTPase, Dyn2, which induces scission of the pit [84] to be posteriorly transformed into early endosomes [25, 27, 77, 78, 86].

Vesicle formation results in spatial and temporal compartmentalization that is controlled by Rab proteins, members of the small GTPase family, which are involved in transmitting signals and providing the identity of the endosome. Tf-TfR complexes accumulate in early endosomes that are specifically marked with Rab5, early endosome antigen 1 (EEA1) [25, 87], and phosphatidylinositol-3,4,5-triphosphate (PI-3,4,5-P3) [26, 75, 88, 89]. Later, complexes are transported to endocytic recycling compartments (ERCs), which present Rab4 [90] and Rab11 [91], where apoTf-TfR and other recycling proteins are concentrated. ERCs are concentrated in close proximity to the nucleus and around the microtubule-organizing center [92, 93].

Actin regulators [92] and other proteins that function in membrane tubulation and fission [94], together with microtubules, are involved in endosome and ERC transportation [95, 96]. Also, specific vesicle-associated membranes (SNARE) proteins that mediate vesicle fusion [97] are important for binding membranes from different vesicles.

In* T. brucei*, the Tf-*Tb*TfR complex is endocytosed in clathrin-coated vesicles [45], and its adaptor protein,* Tb*EpsinR, instead of AP-2 from mammals, promotes clathrin assembly. Additionally, in trypanosomes the endocytosis and scission of the clathrin-coated pits are independent of dynamin [98] (Figure 1, (A)).

Endocytosis in unicellular parasitic protozoa is regulated by the Rab family of proteins. In* T. cruzi,* Tf is transported to reservosomes, structures that are similar to late endosomes, which present* Tc*RAb11 [54]. In* T. brucei,* the* Tb*TfR is recycled back to the flagellar pocket [99] in a recycling system that involves two isoforms of Rab5 (*Tb*Rab5A and* Tb*Rab5B), and* Tb*Rab11 [100]. A similar process occurs in* T. cruzi* and* Leishmania*, where Rab5, identified based on its homology with* Tb*Rab5 [101] and Rab11, may be participating in the recycling of receptors [102], but it is not known in which kind of endosomes they are present exactly. Tf is transported to lysosomes for degradation. Interestingly, in the procyclic stage of* T. brucei *in the invertebrate host, the two Rab5 isoforms occupy the same compartment and have similar effects but in the fluid-phase endocytosis. The differences in endocytic regulation between the two stages of the parasite life cycle show the different mechanisms for surviving in two different hosts: insects and mammals [103, 104].

In the parasite* E. histolytica*,* Eh*Rab11A and* Eh*Rab11B may be participating in the recycling of receptors, because these Rab11s are observed in cells during Fe starvation conditions and in the beginning of the encystation process [105–108]; in addition, these* Eh*Rab11s participate in secretion of cysteine proteases [109].

In* Leishmania*, Rab7 protein promotes fusion with the late endosome during trafficking [110], but* Ld*Rab7 is present in Golgi cisternae, and* E. histolytica Eh*Rab7A is found in endosomes [111]. In the latter protozoan, a genetic screen established the presence of more than 100 Rabs [112], 75% of which are unique to the genus and called RabX, such as* Eh*RabX3 [113], for which the crystallization and preliminary X-ray diffraction analysis were performed. Despite the differences observed, Rab-mediated vesicular trafficking is a well-conserved process in parasitic protozoa.

### 5.2. Inositol-1,4,5-Triphosphate and Diacylglycerol Signaling Pathway

The inositol-1,4,5-triphosphate and diacylglycerol signaling pathway is another important signaling pathway activated by Tf internalization. In this pathway, Ca^2+^ signaling plays a key role in controlling the process of cell proliferation. Tf bound to TfR, a type of tyrosine kinase-linked receptor, stimulates the formation of inositol-1,4,5-triphosphate (InsP_3_) and diacylglycerol (DAG) through the hydrolysis of phosphatidylinositol-4,5-diphosphate (PI-4,5-P2) by phospholipase C (PLC). The released DAG has an important role activating protein kinase C (PKC) and the InsP_3_ diffuses into the cytosol to activate InsP_3_ receptors to release Ca^2+^ stored in the endoplasmic reticulum. The InsP_3_/Ca^2+^ signaling system controls many different cellular processes, such as proliferation [114–116].

This kind of signaling pathway has been described only in* T. brucei *and* Leishmania*. In these parasites, Tf internalization is specifically regulated by glycosylphosphatidylinositol-phospholipase C (GPI-PLC). This enzyme is expressed in the bloodstream form of* T. brucei*. During transformation to the insect stage, GPI-PLC contributes to the release of VSG from the plasma membrane. A new function of the enzyme has been described as a signaling protein that stimulates endocytosis. Similar to that observed in mammalian cells, the products of the enzyme activity are DAG and inositolphosphoglycans (IPG). DAG regulation of Tf internalization depends on proteins with specific domains to act as DAG receptors with protein tyrosine kinase (PTK) and ubiquitin ligase domains [117]. Through the PTK domain, Tf endocytosis is regulated by phosphorylation of the components of the endocytic machinery, such as clathrin, actin, or SNARE proteins. In these organisms, phosphorylation depends on PTKs rather than the Ser/Thr kinases (PKCs) present in vertebrates [117, 118] (Figure 1, (B)).

### 5.3. MAPK Signaling Pathway

Early endosomes and ERCs function as structures for protein assembly in this signaling pathway. The classical example of a protein phosphorylation cascade, highly conserved in eukaryotic organisms, is the mitogen-activated protein kinase (MAPK) pathway that consists of activation of tyrosine kinase-linked receptors, resulting in the phosphorylation of extracellular signal-regulated kinases 1/2 (ERK1/2) that then translocate into the nucleus. This pathway often begins with Ras, another member of the small GTPase family, and its function is the control of many cellular processes, particularly those related to cell proliferation.

Variability in the levels of expression or activity of MAPKs has been correlated with the proliferation, development, or cell cycle progression of many protozoan parasites. Along these lines,* T. brucei *and* Leishmania* MAPKs have been described: mitogen-activated protein kinase (LMPK) of* L. mexicana* [119] and* Tb*MAPK2 of* T. brucei* [39]. In the case of* E. histolytica*, two components of the MAPK signaling pathway have been identified in the* E. histolytica* genome [120]. MAPK belongs to the extracellular signal-regulated kinase (ERK) family, so it may conserve its biological role in regulating the response to the environment for cell proliferation [121] (Figure 1, (C)).

### 5.4. Growth Factors Signaling Pathway

This signaling pathway operates through phosphatidylinositol 3-kinase (PI3K) or PI4K and PI-related kinases with some functions in regulating cell proliferation, such as apoptosis, mitosis, cytokinesis, membrane trafficking, and cytoskeletal organization. The most important component, PI3K, generates the second messenger phosphatidylinositol-3,4,5-trisphosphate (PI3P) that in turn activates both phosphoinositide-dependent kinase 1 (PDK1) and protein kinase B (PKB) that translocate into the nucleus. PI3K confers the mobility needed for clathrin-coated membranes through the microtubule motor machinery [116, 122].

In this sense, PI3K contributes to sending information to the target of rapamycin (TOR) signaling pathway, named because it is inhibited by the drug rapamycin, which is a potent inhibitor of cell proliferation. TOR is a serine/threonine protein kinase, which operates as a nutrient-sensitive cell cycle checkpoint controlling protein synthesis. The activity of TOR is switched off and cell proliferation ceases under conditions of low concentrations of amino acids or when energy is limiting. This kinase is organized into two complexes, TOR1 and TOR2.

A genetic screen of the* T. brucei*,* T. cruzi*,* Leishmania major*,* L. braziliensis*,* L. infantum*, and* E. histolytica* genomes established the presence of several PIKs and PI3Ks, so they have been proposed as a novel signaling pathway [58, 112].

In the kinase TOR, the functions are well conserved in eukaryotes with some differences in cellular localization. The presence of the TOR1 and TOR2 complexes in* T. brucei* and* T. cruzi* was described, and cellular localization was determined in order to define the function, because the localization of signaling molecules is related to their function and specificity. In* T. brucei*,* Tb*TOR1 was observed inside the nucleus and* Tb*TOR2 was associated with the endoplasmic reticulum and mitochondria. In* T. cruzi*,* Tc*TOR1 was absent from the nucleus and was observed close to reservosomes, and* Tc*TOR2 was found dispersed in the cytosol around* Tc*TOR1. These differences in localization suggest a new function of the TOR complex as a result of the high genome plasticity observed in* T. cruzi* originating from different events of intragenic recombination [123]. Different localization could suggest new functions of TOR complexes (Figure 1, (D)).

Several PI3Ks [124, 125], 307 putative PK [120], or hybrid kinases [126], more than 43 putative tyrosine kinases-linked receptors [120], and transmembrane kinases receptors (TMKs) that mediate responses to environment and immune evasion [127, 128] were identified in* E. histolytica*. Unfortunately, the role of these proteins in the endocytosis of Tf and signaling pathways has not been studied.

Further studies are necessary to comprehend the role of these proteins in order to understand the Fe acquisition system of Tf and Fe metabolism in these important parasites.

## 6. Conclusion

The importance of effective Fe uptake has been demonstrated for virulence in several pathogens, and although substantial progress has been made, there is surprisingly little information available about the signal transduction pathways induced by Tf endocytosis in order to obtain Fe. Although the broad picture suggests similarities with the mammalian host, there are many gaps in our understanding of these processes. The identification of signaling proteins will be useful to identify new factors that are essential for parasite adaptation to the host environment.

To date, it has been difficult to compare signal transduction processes in the studied organisms, but it is possible that they are very similar, and as was observed, this similitude may be conserved in intra- and extracellular parasites, despite the fact that they confront Fe absence in different ways.

The differences observed between several proteins and their equivalents in mammals could be used as therapeutic targets that may help treat diseases produced by these parasites, which has implications for biomedical research to develop new chemotherapeutic strategies.
